# Tris{2-[(2,6-dimethyl­phen­yl)amino]­eth­yl}amine

**DOI:** 10.1107/S1600536811049397

**Published:** 2011-11-25

**Authors:** Yurii S. Moroz, Michael K. Takase, Peter Müller, Elena V. Rybak-Akimova

**Affiliations:** a Department of Chemistry, Tufts University, 62 Talbot Avenue, Medford, MA 02155, USA; b Department of Chemistry, Massachusetts Institute of Technology, 77 Massachusetts Avenue, Cambridge, MA 02139, USA

## Abstract

The title compound, C_30_H_42_N_4_, is an aryl­ated tris­(amino­eth­yl)amine derivative which was obtained by reducing the corresponding tris-amide with AlH_3_. The asymmetric unit consists of one third of a *C*_3*v*_-symmetric mol­ecule with the tertiary N atom lying on a crystallographic threefold axis.

## Related literature

For the structural parameters of aryl­ated derivatives of tris­(amino­eth­yl)amine, see: Almesåker *et al.* (2009 ▶); Amoroso *et al.* (2009 ▶). For the synthesis and the structural parameters of metal complexes based on aryl­ated derivatives of tris­(amino­eth­yl)amine, see: Morton *et al.* (2000 ▶); Yandulov & Schrock (2005 ▶); Smythe *et al.* (2006 ▶); Reithofer *et al.* (2010 ▶); Almesåker *et al.* (2010 ▶).
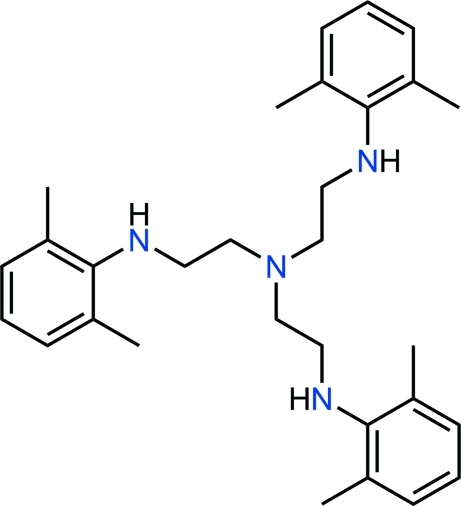


## Experimental

### 

#### Crystal data

C_30_H_42_N_4_
*M* *_r_* = 458.68
Trigonal, 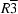

*a* = 14.2880 (7) Å
*c* = 22.3811 (11) Å
*V* = 3956.9 (5) Å^3^
*Z* = 6
Mo *K*α radiation
μ = 0.07 mm^−1^
*T* = 100 K
0.1 × 0.1 × 0.1 mm

#### Data collection

Bruker SMART APEXII CCD diffractometer
Absorption correction: multi-scan (*SADABS*; Sheldrick, 2009 ▶) *T*_min_ = 0.680, *T*_max_ = 0.746
20390 measured reflections
2695 independent reflections
2330 reflections with *I* > 2σ(*I*)
*R*_int_ = 0.031

#### Refinement

*R*[*F* ^2^ > 2σ(*F* ^2^)] = 0.041
*wR*(*F* ^2^) = 0.113
*S* = 1.06
2695 reflections
109 parameters
H atoms treated by a mixture of independent and constrained refinement
Δρ_max_ = 0.42 e Å^−3^
Δρ_min_ = −0.18 e Å^−3^

### 

Data collection: *APEX2* (Bruker, 2009 ▶); cell refinement: *SAINT* (Bruker, 2009 ▶); data reduction: *SAINT*; program(s) used to solve structure: *SHELXS97* (Sheldrick, 2008 ▶); program(s) used to refine structure: *SHELXL97* (Sheldrick, 2008 ▶); molecular graphics: *ORTEP-3 for Windows* (Farrugia, 1997 ▶); software used to prepare material for publication: *WinGX* (Farrugia, 1999 ▶).

## Supplementary Material

Crystal structure: contains datablock(s) I, global. DOI: 10.1107/S1600536811049397/zl2430sup1.cif

Structure factors: contains datablock(s) I. DOI: 10.1107/S1600536811049397/zl2430Isup2.hkl

Supplementary material file. DOI: 10.1107/S1600536811049397/zl2430Isup3.cml

Additional supplementary materials:  crystallographic information; 3D view; checkCIF report

## Figures and Tables

**Fig. 1.**
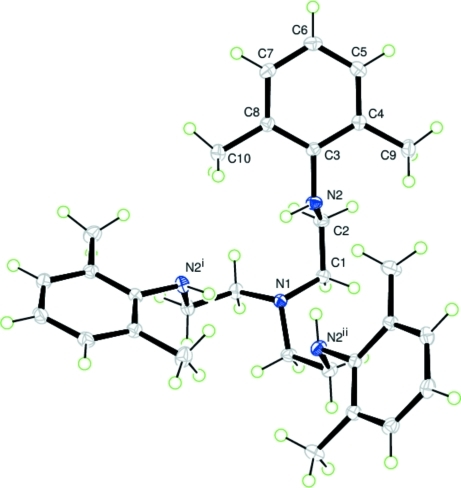
A view of the title compound, with displacement ellipsoids shown at the 50% probability level. Symmetry transformations used to generate equivalent atoms: (i) -y+1, x-y, z; (ii) -x+y+1, -x+1, z.

**Fig. 2.**
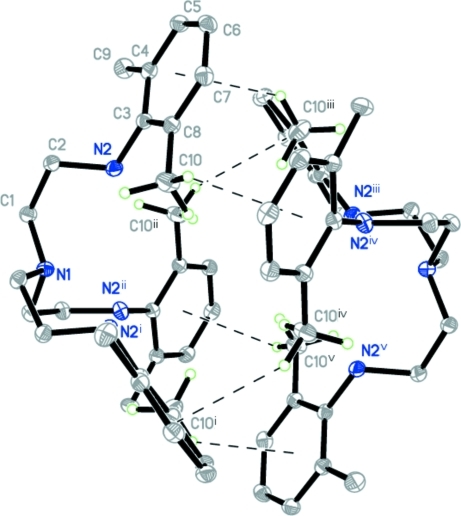
A fragment of the packing diagram of the title compound, with displacement ellipsoids shown at the 50% probability level (H atoms, except H atoms attached to C10 atom, are omitted for clarity). Symmetry transformations used to generate equivalent atoms: (i) -y+1, x-y, z; (ii) -x+y+1, -x+1, z; (iii) 1/3+x-y, -1/3+x, 2/3-z; (iv) 1/3+y, 2/3-x+y, 2/3-z; (v) 1/3-x, 2/3-y, 2/3-z.

## supplementary crystallographic information

### Comment

Tris(aminoethyl)amine derivatives have attracted attention of chemists due to their ability to adopt a trigonal pyramidal geometry which is favourable for coordination of different metal ions in a trigonal bipyramidal environment, with one open coordination site for a small exchangeable ligand (Morton *et al.*, 2000; Yandulov *et al.*, 2005; Smythe *et al.*, 2006; Reithofer *et al.*, 2010; Almesåker *et al.*, 2010). In this report, we disscuss the molecular structure of an arylated tris(aminoethyl)amine derivative which appears to be a promising ligand for obtaining high valent iron compounds.

The title compound (1) crystallizes in the trigonal space group *R*3 and consists of neutral molecules (Figure 1); inter-molecular interactions include a number of van der Waals and C–H···π contacts. There are two types of the C—H···π contacts that originate from hydrogen atoms of the methyl groups pointing towards the opposite sides of the same aromatic ring; no aryl H atoms are involved. The first type of non-covalent interactions has a C10 atom acting as a donor (the C—H···π separation is 3.530 (1) A) and results in the formation of pseudo-dimer aggregates (Figure 2) which form a three-dimensional, well defined symmetric cavity *via* the second type of C—H···π contacts and van der Waals contacts. The second type of C–H···π contacts includes C9 as a donor (the C–H···π separation is 3.641 (1) Å).

The secondary amino group is located in a *cis*-position to the tertiary N atom (N1—C1—C2—N2 torsion angle is 54.0 (1)°). The C—C, C—N bond lengths are comparable to the previously reported structures of arylated derivatives of tris(aminoethyl)amine (Almesåker *et al.*, 2009; Amoroso *et al.*, 2009).

### Experimental

The title compound, (1), was obtained in three steps. Nitrilotriacetoanilide, (ArNC(O)CH_2_)_3_N, where Ar = Me_2_C_6_H_3_, was synthesized *via* the reaction of nitrilotriacetic acid chloride and 2,6-dimethylaniline. The acid chloride was prepared *in situ*: Oxalyl chloride (10.6 ml) was added dropwise to a cooled (278 K, 5 °C) mixture of nitrilotriacetic acid (5 g, 0.03 mol, in 100 ml of DCM) with one drop of DMF as a catalyst. The mixture was stirred for 48 h at room temperature, and then the DCM and extra oxalyl chloride were removed by vacuum distillation. The crude acid chloride was dissolved in 50 ml of DCM and added dropwise to a 100 ml of DCM solution of 2,6-dimethylaniline (9.8 ml, 0.08 mol) and *N*-ethyldiisopropylamine (18.5 ml, 0.11 mol) at 263 K (–10 °C). After the addition was complete, the reaction mixture was allowed to warm up and stirred for 24 h at ambient temperature. The reaction mixture was washed with 1 N HCl (25 ml), and then with saturated NaHCO_3_ (25 ml). The organic layer was dried (Na_2_SO_4_) and concentrated under reduced pressure. The solid was washed with water/methanol, 1/1 (*v*/*v*), filtered, and dried in an oven at 373 K (100 °C) for 2 days. Yield: 3.07 g (23%). ^1^H NMR (300 MHz, dmso-*d*_6_): δ 2.16 (s, 18, Me), 3.70 (s, 6, CH_2_), 7.08 (m, 9, H_p_, 2H_m_), 9.63 (s, 3, NH). ^13^C NMR (75 MHz, dmso-*d*_6_): δ 18.21, 57.99, 126.6, 127.74, 134.86, 135.21, 168.82.

*N*^1^,*N*^2^,*N*^3^-Tris((2,6-dimethylphenyl)amino)ethyl)amine: To 200 ml of dry THF, 7.20 g (0.2 mol) of LiAlH_4_ was added slowly in portions. Then the reaction mixture was cooled in an ice bath and 26 ml (0.2 mol) of chlorotrimethylsilane was added dropwise, followed by an addition of 3.07 g (0.006 mol) of nitrilotriacetoanilide. The reaction mixture was refluxed for 14 h (the reaction was controlled by NMR) and then cooled down to room temperature. Then 21 ml of water in 40 ml of THF was carefully added to the reaction mixture, followed by the addition of NaOH (50%, 21 ml). The reaction mixture was filtered, the precipitate was washed with THF (100 ml) and the filtrate was evaporated under reduced pressure. The solid was extracted with DCM (100 ml); the DCM solution was dried (Na_2_SO_4_) and concentrated. The crude product was washed with cold diethyl ether (100 ml), filtered, and dried under reduced pressure. Yield: 1.5 g (54%). Colourless crystals, which were suitable for X-ray analysis, were grown in an NMR tube from the dmso-*d*_6_ solution. ^1^H NMR (300 MHz, dmso-*d*_6_): δ 2.18 (s, 18, Me), 2.64 (t, *J* = 6.3 Hz, 6, CH_2_), 2.99 (td, *J* = 6.3, 6 Hz, 6, CH_2_), 3.83 (t, *J* = 6 Hz, 3, NH), 6.69 (t, *J* = 7.2 Hz, 3, H_p_), 6.90 (d, *J* = 7.2 Hz, 6, H_m_). ^13^C NMR (75 MHz, dmso-*d*_6_): δ 18.47, 45.54, 54.51, 120.8, 128.51, 146.38.

### Refinement

All methyl H atoms were placed in geometrically idealized positions, allowing the initial torsion angle to be determined by a difference Fourier analysis and subsequently refined [C—H = 0.98 Å and *U*_iso_(H) = 1.5 *U*_eq_(C)]. Other H atoms bonded to C atoms were placed in geometrically idealized positions and included as riding atoms [C—H = 0.95–0.99 Å and *U*_iso_(H) = 1.2 *U*_eq_(C)]. The position and *U*_iso_ value of H atom bonded to N atom were fully refined. The highest peak is located 0.75 Å from atom C2 and the deepest hole is located 1.26 Å from atom C6.

### Crystal data

**Table:**

C_30_H_42_N_4_	*D*_x_ = 1.155 Mg m^−^^3^
*M**_r_* = 458.68	Mo *K*α radiation, λ = 0.71073 Å
Trigonal, *R*3	Cell parameters from 9944 reflections
Hall symbol: -R 3	θ = 2.5–30.6°
*a* = 14.2880 (7) Å	µ = 0.07 mm^−^^1^
*c* = 22.3811 (11) Å	*T* = 100 K
*V* = 3956.9 (5) Å^3^	Block, colourless
*Z* = 6	0.1 × 0.1 × 0.1 mm
*F*(000) = 1500	

### Data collection

**Table:**

Bruker Smart APEXII CCD diffractometer	2695 independent reflections
Radiation source: ImuS micro-focus sealed tube	2330 reflections with *I* > 2σ(*I*)
Icoatech ImuS multilayer optics	*R*_int_ = 0.031
Detector resolution: 8.3 pixels mm^-1^	θ_max_ = 30.6°, θ_min_ = 1.9°
φ and ω scans	*h* = −20→20
Absorption correction: multi-scan (*SADABS*; Sheldrick, 2009)	*k* = −20→20
*T*_min_ = 0.680, *T*_max_ = 0.746	*l* = −31→31
20390 measured reflections	

### Refinement

**Table:**

Refinement on *F*^2^	Primary atom site location: structure-invariant direct methods
Least-squares matrix: full	Secondary atom site location: difference Fourier map
*R*[*F*^2^ > 2σ(*F*^2^)] = 0.041	Hydrogen site location: inferred from neighbouring sites
*wR*(*F*^2^) = 0.113	H atoms treated by a mixture of independent and constrained refinement
*S* = 1.06	*w* = 1/[σ^2^(*F*_o_^2^) + (0.0529*P*)^2^ + 4.1067*P*] where *P* = (*F*_o_^2^ + 2*F*_c_^2^)/3
2695 reflections	(Δ/σ)_max_ < 0.001
109 parameters	Δρ_max_ = 0.42 e Å^−^^3^
0 restraints	Δρ_min_ = −0.18 e Å^−^^3^

### Special details

**Table:**

Geometry. All s.u.'s (except the s.u. in the dihedral angle between two l.s. planes) are estimated using the full covariance matrix. The cell s.u.'s are taken into account individually in the estimation of s.u.'s in distances, angles and torsion angles; correlations between s.u.'s in cell parameters are only used when they are defined by crystal symmetry. An approximate (isotropic) treatment of cell s.u.'s is used for estimating s.u.'s involving l.s. planes.
Refinement. Refinement of *F*^2^ against ALL reflections. The weighted *R*-factor *wR* and goodness of fit *S* are based on *F*^2^, conventional *R*-factors *R* are based on *F*, with *F* set to zero for negative *F*^2^. The threshold expression of *F*^2^ > 2σ(*F*^2^) is used only for calculating *R*-factors(gt) *etc*. and is not relevant to the choice of reflections for refinement. *R*-factors based on *F*^2^ are statistically about twice as large as those based on *F*, and *R*- factors based on ALL data will be even larger.

### Fractional atomic coordinates and isotropic or equivalent isotropic displacement parameters (Å^2^)

**Table:**

	*x*	*y*	*z*	*U*_iso_*/*U*_eq_	
C1	0.58018 (7)	0.22760 (7)	0.11134 (4)	0.01668 (18)	
H1A	0.5860	0.2233	0.0675	0.020*	
H1B	0.5095	0.2218	0.1200	0.020*	
C2	0.58289 (8)	0.13268 (7)	0.13995 (4)	0.01696 (18)	
H2A	0.5189	0.0642	0.1271	0.020*	
H2B	0.6485	0.1316	0.1268	0.020*	
C3	0.58132 (7)	0.05566 (7)	0.23806 (4)	0.01446 (17)	
C4	0.48468 (7)	−0.04409 (7)	0.24161 (4)	0.01604 (18)	
C5	0.48377 (8)	−0.12750 (8)	0.27456 (4)	0.01872 (19)	
H5	0.4192	−0.1956	0.2766	0.022*	
C6	0.57519 (8)	−0.11311 (8)	0.30435 (4)	0.01925 (19)	
H6	0.5732	−0.1708	0.3264	0.023*	
C7	0.66954 (8)	−0.01359 (8)	0.30156 (4)	0.01794 (18)	
H7	0.7318	−0.0032	0.3225	0.022*	
C8	0.67430 (7)	0.07137 (7)	0.26845 (4)	0.01593 (18)	
C9	0.38314 (8)	−0.06114 (8)	0.21179 (5)	0.0221 (2)	
H9A	0.3838	−0.0794	0.1696	0.033*	
H9B	0.3791	0.0052	0.2146	0.033*	
H9C	0.3202	−0.1203	0.2316	0.033*	
C10	0.77793 (8)	0.17807 (8)	0.26578 (5)	0.0238 (2)	
H10A	0.8351	0.1721	0.2866	0.036*	
H10B	0.7675	0.2338	0.2850	0.036*	
H10C	0.7988	0.1979	0.2240	0.036*	
N1	0.6667	0.3333	0.13202 (6)	0.0142 (2)	
N2	0.58312 (7)	0.14184 (6)	0.20523 (4)	0.01647 (17)	
H2N	0.6392 (12)	0.2043 (12)	0.2164 (6)	0.024 (3)*	

### Atomic displacement parameters (Å^2^)

**Table:**

	*U*^11^	*U*^22^	*U*^33^	*U*^12^	*U*^13^	*U*^23^
C1	0.0167 (4)	0.0151 (4)	0.0166 (4)	0.0067 (3)	−0.0036 (3)	−0.0003 (3)
C2	0.0202 (4)	0.0147 (4)	0.0158 (4)	0.0086 (3)	−0.0020 (3)	−0.0008 (3)
C3	0.0160 (4)	0.0149 (4)	0.0142 (4)	0.0090 (3)	−0.0006 (3)	−0.0009 (3)
C4	0.0154 (4)	0.0168 (4)	0.0159 (4)	0.0081 (3)	−0.0001 (3)	−0.0016 (3)
C5	0.0195 (4)	0.0153 (4)	0.0201 (4)	0.0077 (3)	0.0034 (3)	0.0007 (3)
C6	0.0240 (4)	0.0184 (4)	0.0196 (4)	0.0137 (4)	0.0037 (3)	0.0036 (3)
C7	0.0191 (4)	0.0216 (4)	0.0173 (4)	0.0133 (4)	−0.0003 (3)	0.0011 (3)
C8	0.0155 (4)	0.0164 (4)	0.0160 (4)	0.0081 (3)	−0.0008 (3)	−0.0007 (3)
C9	0.0149 (4)	0.0237 (5)	0.0238 (5)	0.0068 (4)	−0.0031 (3)	−0.0002 (4)
C10	0.0176 (4)	0.0201 (4)	0.0286 (5)	0.0055 (4)	−0.0063 (4)	0.0024 (4)
N1	0.0130 (3)	0.0130 (3)	0.0167 (6)	0.00651 (17)	0.000	0.000
N2	0.0204 (4)	0.0139 (3)	0.0158 (4)	0.0091 (3)	−0.0030 (3)	−0.0011 (3)

### Geometric parameters (Å, °)

**Table:**

C1—N1	1.4686 (10)	C6—C7	1.3879 (14)
C1—C2	1.5178 (12)	C6—H6	0.9500
C1—H1A	0.9900	C7—C8	1.3946 (12)
C1—H1B	0.9900	C7—H7	0.9500
C2—N2	1.4667 (12)	C8—C10	1.5043 (13)
C2—H2A	0.9900	C9—H9A	0.9800
C2—H2B	0.9900	C9—H9B	0.9800
C3—C4	1.4059 (12)	C9—H9C	0.9800
C3—C8	1.4069 (12)	C10—H10A	0.9800
C3—N2	1.4231 (11)	C10—H10B	0.9800
C4—C5	1.3961 (13)	C10—H10C	0.9800
C4—C9	1.5020 (13)	N1—C1^i^	1.4686 (10)
C5—C6	1.3872 (14)	N1—C1^ii^	1.4686 (10)
C5—H5	0.9500	N2—H2N	0.886 (15)
			
N1—C1—C2	113.69 (7)	C6—C7—C8	121.01 (9)
N1—C1—H1A	108.8	C6—C7—H7	119.5
C2—C1—H1A	108.8	C8—C7—H7	119.5
N1—C1—H1B	108.8	C7—C8—C3	119.13 (8)
C2—C1—H1B	108.8	C7—C8—C10	119.87 (8)
H1A—C1—H1B	107.7	C3—C8—C10	120.99 (8)
N2—C2—C1	109.91 (7)	C4—C9—H9A	109.5
N2—C2—H2A	109.7	C4—C9—H9B	109.5
C1—C2—H2A	109.7	H9A—C9—H9B	109.5
N2—C2—H2B	109.7	C4—C9—H9C	109.5
C1—C2—H2B	109.7	H9A—C9—H9C	109.5
H2A—C2—H2B	108.2	H9B—C9—H9C	109.5
C4—C3—C8	120.33 (8)	C8—C10—H10A	109.5
C4—C3—N2	119.34 (8)	C8—C10—H10B	109.5
C8—C3—N2	120.30 (8)	H10A—C10—H10B	109.5
C5—C4—C3	118.71 (8)	C8—C10—H10C	109.5
C5—C4—C9	120.02 (8)	H10A—C10—H10C	109.5
C3—C4—C9	121.26 (8)	H10B—C10—H10C	109.5
C6—C5—C4	121.41 (9)	C1^i^—N1—C1	110.54 (6)
C6—C5—H5	119.3	C1^i^—N1—C1^ii^	110.54 (6)
C4—C5—H5	119.3	C1—N1—C1^ii^	110.54 (6)
C5—C6—C7	119.38 (9)	C3—N2—C2	116.04 (7)
C5—C6—H6	120.3	C3—N2—H2N	110.0 (9)
C7—C6—H6	120.3	C2—N2—H2N	109.3 (9)
			
N1—C1—C2—N2	54.02 (10)	C6—C7—C8—C10	−179.46 (9)
C8—C3—C4—C5	−1.51 (13)	C4—C3—C8—C7	0.65 (13)
N2—C3—C4—C5	−179.24 (8)	N2—C3—C8—C7	178.35 (8)
C8—C3—C4—C9	177.26 (8)	C4—C3—C8—C10	−179.19 (9)
N2—C3—C4—C9	−0.47 (13)	N2—C3—C8—C10	−1.48 (14)
C3—C4—C5—C6	1.07 (14)	C2—C1—N1—C1^i^	67.79 (13)
C9—C4—C5—C6	−177.72 (9)	C2—C1—N1—C1^ii^	−169.49 (8)
C4—C5—C6—C7	0.25 (14)	C4—C3—N2—C2	−74.71 (11)
C5—C6—C7—C8	−1.16 (14)	C8—C3—N2—C2	107.56 (10)
C6—C7—C8—C3	0.70 (14)	C1—C2—N2—C3	177.64 (7)

Symmetry codes: (i) −*y*+1, *x*−*y*, *z*; (ii) −*x*+*y*+1, −*x*+1, *z*.
